# The Healthy Start scheme in England “is a lifeline for families but many are missing out”: a rapid qualitative analysis

**DOI:** 10.1186/s12916-024-03380-5

**Published:** 2024-05-08

**Authors:** Millie Barrett, Mark Spires, Christina Vogel

**Affiliations:** 1 Centre for Food Policy, City, University of London, London, UK; 2 Medical Research Council Lifecourse Epidemiology Centre, University of Southampton, Southampton General Hospital, Tremona Road, Southampton, UK; 3 National Institute for Health and Care Research Southampton Biomedical Research Centre, University of Southampton and University Hospital Southampton NHS Foundation Trust, Tremona Road, Southampton, UK

**Keywords:** Food subsidy programme, Healthy Start, Rapid qualitative analysis, Systems approach, Dietary inequalities

## Abstract

**Background:**

Healthy Start (HS) is a government scheme in England, Wales and Northern Ireland that offers a financial payment card and free vitamins to families experiencing low income. Pregnant women and families with children < 4 years can use the HS card to buy fruit, vegetables, cow’s milk, infant formula and pulses. HS was fully digitalised in March 2022. While digitalisation has improved the user experience for many families, in the context of the cost-of-living crisis and increasing dietary inequalities, it is important to understand why HS is not reaching more families. This study aimed to (i) assess the perceptions and experiences of HS from stakeholders across the system including those who promote, implement and are eligible for HS, and (ii) identify recommendations to improve the scheme’s effectiveness and uptake.

**Methods:**

The study design was a post-implementation rapid qualitative evaluation using stakeholder interviews. Data were collected between January and June 2023 via semi‐structured interviews (50% online; 50% in person) with 112 stakeholders, including parents (*n* = 59), non‐government organisations (*n* = 13), retailers (*n* = 11) and health and community professionals (*n* = 29) at national and local levels. Findings were confirmed by a sub-sample of participants.

**Results:**

Six core themes cut across stakeholders’ perceptions and experiences, and stakeholders collectively outlined seven recommendations they felt could be acted upon to maximise uptake and efficiency of HS, with actions at both national and local levels. A novel finding from this study is that raising awareness about HS alone is unlikely to result automatically or universally in higher uptake rate. Recommendations include: continuing to provide this scheme that is universally valued; the need for many families to be provided with a helping hand to successfully complete the application; reframing of the scheme as a child’s right to food and development to ensure inclusivity; improved leadership, coordination and accountability at both national and local levels.

**Conclusions:**

HS provides benefits for child development and family wellbeing. The study’s recommendations should be actioned by national and local governments to enable all families eligible for the scheme to benefit from this nutritional safety net.

**Supplementary Information:**

The online version contains supplementary material available at 10.1186/s12916-024-03380-5.

## Background

Food insecurity is defined as a lack of regular access to enough, safe and healthy nutritious food for normal growth and development and an active, healthy life [1]. In June 2023 almost one quarter of UK households with children reported experiencing food insecurity [2] and families with pre-school aged children are more likely to experience food insecurity than other families. The cost-of-living crisis has seen the percentage of families with children experiencing moderate or severe food insecurity double between January 2022 and August 2023 [2]. Inflation is affecting the ability of families experiencing low income to afford the basics including rent, utility bills and food [3]. Food prices have risen rapidly since the middle of 2021 [3]. Families with the lowest incomes spend a greater proportion of their household budget on food than those with higher incomes [4] and are therefore more affected by food inflation. Food insecure households are more likely to cut back on healthy foods including fruit, vegetables and dairy [2]. With healthy foods costing more than unhealthy foods and being less desirable due to perishability and household food preferences [5, 6], families experiencing lower incomes are driven towards opting for cheap, ultra-processed, nutrient-poor products [7, 8].

Having to eat poor-quality food leads to malnutrition and is a key reason why obesity is aligned with socioeconomic deprivation. Children in the UK who are born and grow up in families experiencing low income are exposed to many more social and environmental risk factors than protective factors for poor diet and unhealthy weight gain [9]. Continual exposure to unhealthy food environments leads to obesity among children living in poorer families. Children living in more affluent families are shielded by many more protective factors in their social and physical environments [9, 10].

Food insecurity and poor diet in early life detrimentally affects a person’s physical and mental health, and later life educational and employment opportunities [11, 12]. In recognition of this evidence, the UK government’s Healthy Start scheme (HS) offers families experiencing low-income financial support to buy healthy foods [13]. HS was established in 2006 and is available to pregnant women and families with children under the age of 4 years with very low incomes. All pregnant women under 18 years can also access HS. When initially implemented, the scheme provided £2.80 per week per child to purchase fresh fruit, vegetables, cow’s milk and infant formula. The value increased to £3.10 in 2009. The range of products that could be purchased was expanded in 2011 to include frozen fruit and vegetables, then again in 2020 to include pulses and beans, as well as canned fruit and vegetables (without additional fat, salt, sugar or flavouring). In April 2021, the weekly value increased to £4.25 per week. The value of HS for infants aged 0–12 months has always been double the baseline amount, thus now providing £8.50 per week.

Prior assessments of HS have indicated that families value the scheme because it allows them to purchase more fruit, vegetables and milk [5, 6, 14, 15], or buy other essential items with the freed-up money [[5, 6, 14–16]. The paper-based voucher system, however, left some families feeling stigmatised and required retailers to register and claim for reimbursement [6]. By January 2020, uptake of HS in England had dropped to around 50% of eligible families. HS was fully digitalised in April 2022 to make the scheme easier for families and retailers. A payment card (Mastercard™) is now issued by service provider NHS Business Services Authority (NHSBSA) to eligible families following a successful online application and is topped up each month according to the number of eligible children in the household. Current figures show that uptake has increased, but 34% of eligible families in England are still not accessing HS, with approximately 171,000 missing out [17] on a total of £45 m of financial support each year [18]. With dietary inequalities increasing among UK children and the associated long-term health and productivity outcomes being detrimental for British society [19], it is important to understand why more families are not accessing HS.

This study addresses the current evidence gap by conducting a post-implementation rapid qualitative evaluation of the HS scheme, particularly focusing on the payment card. Qualitative methods were employed to examine how this food policy is being implemented and its impact within the surrounding system [20]. Qualitative research methods are useful for exploring system complexity because they allow researchers to delve deeply into the various elements, structures and relationships within the system affected by the policy [21, 22]. This study applied a rapid qualitative research approach [23, 24] to provide unique and timely findings from a large number of participants. Participatory techniques [25] were also employed to validate findings with participants and other stakeholders.

The specific aims of this study were to (i) examine stakeholders’ perspectives and experiences of HS in England and (ii) identify stakeholder recommendations to improve effectiveness and take up of the HS payment card in England.

## Methods

### Study design and setting

The study outlined in this paper is the first stage of a larger mixed-methods evaluation of HS [26]. The setting for this study was England, and the design was a post-implementation rapid qualitative evaluation. The study incorporated both national and local perspectives. National perspectives were obtained from organisations or companies with national reach. Three case study areas were predominantly used to provide the local perspectives, namely the cities of Southampton and Manchester, and the London Borough of Redbridge. These locations were purposively selected because they all have child poverty rates above the national average of 31% (Southampton 34%, Redbridge 40% and Manchester 42% [59]), they offer culturally and regionally diverse perspectives across England. In addition, the three study sites were selected to include areas with average (Southampton), above average (Manchester) and below average (Redbridge) uptake of HS. Local perspectives were also derived from a small number of localities (*n* = 8) outside of these three areas to provide a wider understanding of local activities related to HS.

Ethical approval for all phases of the study was granted by the Faculty of Medicine Ethics Board at the University of Southampton (ERGO76125) and the School of Health and Psychological Sciences Ethics Committee at City, University of London (ETH223-1264). Compensation of a £15 shopping voucher was provided to all participating parents. The value and form of compensation was agreed with the study’s public contributor panel which includes cultural, regional and gender diverse parents eligible for HS.

### Sampling and data collection

A purposive sample of participants was taken from five main stakeholder groups, namely: (i) families eligible for the scheme; (ii) local authority staff working in the fields of public, maternal and/or child health; (iii) health professionals working in obstetrics and paediatrics; (iv) non-government organisations (NGOs) and charities; and (v) food retailers (national chains, independent retailers and market stalls). Recruitment followed two phases and all data were collected between December 2022 and June 2023. Only families took part in Phase 1 and all stakeholder groups took part in Phase 2. The purpose of Phase 1 was to reach a large and diverse sample of families in each study site and gather rich contextual data using novel research techniques.

#### Phase 1: Shallow dive interviews

Shallow dive interviews were conducted by the social enterprise Activmob who have extensive expertise in engaging harder-to-reach families [27]. Participants were approached and recruited via trusted public locations including children’s centres, community markets, food outlets and community hubs. Potential participants were given an information sheet explaining the study, highlighting that taking part was voluntary and that they could withdraw at any point. Participants were asked to sign a consent form. All participant materials were designed to be accessible to families eligible for HS and were co-designed with the study’s public contributor panel. Participants had no prior relationships with the two female Activmob researchers. A snowball sampling procedure was employed whereby participants could introduce the researchers to their social network. Interviews lasted approximately 30 min and were not recorded to enhance participation. Detailed field notes were recorded by one researcher while another led the interview using a semi-structured topic guide.

#### Phase 2: Semi-structured interviews

Semi-structured interviews with all five stakeholder groups were conducted by MB, with some undertaken jointly with CV. Families, local authority staff, health professionals, NGOs and retailers were recruited via email or in-person using a range of approaches including: (i) existing professional contacts and networks, (ii) desktop identification of stakeholders in the three case study areas and (iii) snowball sampling whereby participants introduced other appropriate contacts to the research team. Participants were sent the study information sheet and consent form for review and completion prior to interview. Participants were given the opportunity to ask questions and/or withdraw. Interviews lasted between 20 and 45 min and were held face-to-face or using video conferencing software (Microsoft Teams). Most interviews were with individuals, but in some cases two members of the same business or organisation took part in a joint interview.

### Interview guides

Semi-structured interview guides were developed for each stakeholder group (See Additional Annex). This approach allows topics to be explored systematically while also allowing participants to raise issues they feel are relevant [28]. Consistent with an inductive approach [29], interviews were designed to be flexible and followed a general topic-oriented structure. All interviews sought to elicit detailed accounts of people’s experiences and perceptions of HS in their capacity of supporting, implementing or being eligible for the scheme.

One interview guide was developed for use with families in both Phase 1 and Phase 2. Families were asked general open-ended questions related to HS. Topics covered included awareness of and access to the scheme, experiences of the scheme pre/post-digitalisation, experiences of add-on services such as retailer top-ups or local group sessions, general feelings about HS and thoughts about how the scheme could be improved. A second interview guide was used with local authority staff, health professionals and NGOs, and a third guide was developed for participants from the retail sector. Topics covered were similar to those discussed with families but were positioned around supporting families to sign up and use the scheme or related to the importance of the scheme to their business, add-on activities employed by their business and any perceived impact on families’ shopping practices. All interview guides ended with an invitation for participants to raise points of importance to them which had not arisen during the interview.

### Data analysis

Rapid qualitative methodology was used to analyse data from both Phase 1 and Phase 2 interviews to enable the research team to collate valid, timely results from a variety of stakeholders. This methodological approach enables data from a large number of participants to be synthesised quickly and is particularly suited for time-sensitive policy studies to allow results to be shared with policymakers quickly [21, 23]. Results from rapid qualitative analyses have been found to be comparable to more established, time-intensive qualitative methods [30, 31]*.*

After each interview, summary notes were produced by a researcher either based on fieldnotes (Phase 1) or while listening back to the recording (Phase 2). Data from each summary in Phase 2 were entered into stakeholder-specific Rapid Assessment sheets (RAP sheets) detailing summary points. Each stakeholder RAP sheet was sectioned into categories labelled (i) benefits of HS, (ii) barriers to HS uptake and (iii) HS promotion and factors to enhance uptake. Under each of these categories, subheadings were allocated to capture key themes relevant across stakeholder groups. This approach allowed common themes to be drawn out and applied across all the RAP sheets to incorporate all stakeholder views. Data from Phase 1 summary notes were added to the families RAP sheets. The RAP sheets constitute the study data [21, 23]. Themes and sub-themes from both Phase 1 and Phase 2 datasets were identified to address the two study aims and were verified through formal dialogues involving MB, CV, MS and the Activmob researchers. Inconsistencies arising during the analytical process were resolved through subsequent dialogue and referring to the study data. Illustrative quotes were retrieved from both Phase 1 and Phase 2 datasets.

MB and CV have expertise in Public Health Nutrition. MS is a male Public Health researcher with expertise in qualitative lived experience research. The two Activmob researchers have expertise in qualitative research methods.

### Validation of themes

Following data analysis, emerging themes and sub-themes were confirmed using participatory methods [25] with key stakeholders representing all five stakeholder groups from the three case study areas as well as the study’s public contributor panel during virtual and in-person meetings. These meetings involved sharing the research team’s preliminary findings and providing an opportunity to confirm and adjust the results. As part of this process, the phrasing of some theme and sub-theme titles were adapted to better reflect stakeholder experiences. In keeping with rapid qualitative research techniques [23, 24], reports and presentations of emerging findings were also shared with UK government policymakers. Through this process, some sub-themes (1.iii, 1.iv, 3.i, 3.iii) were raised as being important to the successful delivery and effectiveness of the HS scheme; however, they fall outside the scope of the commissioned research and will therefore not be examined beyond the current study.

## Results

### Participant profile

A total of 112 participants took part in this study (Table 1). The majority (84%, *n* = 94) of participants were recruited from the local case study areas or other local areas in England with notable activity related to HS. More than half (53%, *n* = 59) were parents with young children experiencing very low annual household income. Most parents (*n* = 48/59) took part in Phase 1 data collection. Across both phases, most parents were mothers (*n* = 53/59). Local authority staff (both managers and frontline staff) contributed to 32% of the sample. The remaining participants represented health professional, NGO, and retailer (including market stalls) stakeholder groups and provide both local and national perspectives on the HS scheme.

**Table 1:** Participant profile according to stakeholder group

Stakeholder group	Local	National
Parents^a^	59	N/A
Local Authority staff *(managers and frontline)*	19	N/A
Health professionals	8	2
NGOs/advocacy groups	5	8
Retailers	3	8
Sub-total	94	18
**Total**	**112**	

^a^48 parents took part in Phase One of the research and were spoken to by Activmob, the remaining parents took part in Phase Two and were interviewed by MB

### Aim 1: To examine stakeholders’ perspectives and experiences of HS in England

Rapid analysis identified six core themes that reflected the perceptions and experiences across all five stakeholder groups. These six themes and their corresponding sub-themes are presented below alongside illustrative verbatim quotes from participants.

#### HS is wanted and needed, especially now

HS is highly valued by stakeholders across the system, including families, professionals and retailers. The scheme can make a tangible difference to household food budgets particularly now when more families with children are experiencing food insecurity than ever before. Some important areas for improvement in the scheme were also identified.

“We only have positive things to say about Healthy Start, our families say they’d be lost without it, they really appreciate it, it makes a huge difference, and families do feel they can take a bit of control, and especially when using foodbanks they can get a lot more, it means the world to families.” Local Authority, ID5038.

##### The payment card is better

Stakeholders across the system agree that the payment card is an improvement on the previous paper voucher scheme.

“Having the digital card now is so much better, I have more flexibility as to where to spend the money, it makes a huge difference. There are no problems using the card and I have much less anxiety.” Mother, ID6005

Although communications about the scheme’s changes were issued directly to families, many struggled to sign up successfully due to lack of digital access or skills, not appreciating the need to reapply, or other problems with transition to the digital system.

“We have high levels of digital exclusion in the two most deprived wards where we work. This was a major issue when the scheme went digital, we have a cohort of families that are particularly hard to reach. They don’t come into the children’s centres, so it was difficult to let them know they needed to reapply and a lot of them probably haven’t done that.” Local Authority, ID5038

##### HS changes how we eat

The HS payments allow families to buy foods they would not otherwise be able to afford, allowing children to be introduced to healthy foods from an early age.

“My children’s diets improved as a direct result of Healthy Start because I always knew I had that extra bit of money each week to get just fruits and milk. Without Healthy Start you have to think ‘maybe this week I can do without’.” Mother, ID6003

Many stakeholders recognised that HS also offers wider benefits for families.

“Healthy Start is not only about food, healthy diet impacts on everything, including mental health and it’s about so much more than food.” Local Authority, ID5008

##### Four‐year‐olds are missing out

Overwhelmingly, families and other stakeholders noted the gap in provision of healthy foods from when a child turns four until they start school and can access other forms of nutritional support. Some families described that by age four their children have developed preferences for healthy foods which then become unaffordable to provide.

“I like to give them fruit and milk every day but some of that will come to an end [now Healthy Start has stopped]. I am thinking carefully about what foods to give each day as now I can’t afford milk and fruit every day. But how do I explain that to my 4‐year‐old?” Mother, ID6005

“The upper age limit should be increased to dovetail with FSM because nutrient requirements don’t end when a child turns 4.” National Health Professional, ID5004

##### The card value covers less now

All stakeholders noted that the value of HS has not kept up with the rising cost of food. While the price of milk, fruit and vegetables have all increased, the rapid rise in cost of infant formula has been particularly difficult for families to manage.

“I don’t want to be ungrateful for the help but after buying enough milk for the baby there is only £3‐4 left over in the month, so I buy veg with the rest. Due to rising cost of milk the value of the card goes a lot less far than before.” Mother, ID6004

“The money is good, but it doesn’t go very far with food inflation and prices as they are now.” National retailer, ID5010

#### Raising uptake is about more than just awareness

It is apparent that knowledge of HS is not ubiquitous across stakeholder groups. However, there are a number of reasons why increasing knowledge or awareness of the scheme alone would not automatically optimise uptake.

##### HS is “not for me”

Among some families, there is a belief that HS is “not for me”. This belief might be driven by a variety of factors such as cultural practices, ideologies, values or sense of identity. Work needs to be done to unravel why some communities and families feel HS is not for them, and to co‐create solutions that allow people who are currently missing out to access the scheme.

“People hear about HS and think ‘that’s not for me’ and we need to do more to understand why that is and what those barriers are for some families.” National NGO, ID5040.

“HS is the white people’s thing, that’s not for us.” Local Authority, ID5033.

“Often it’s the male partner who does the benefits applications and the woman is not involved, so even though she may hear about HS she thinks he is taking care of that side of things.” Local Health Professional, ID5029.

##### Fear of authority

Some families noted that a particular barrier to applying for HS is a fear of the payments affecting their other income streams, or worse, having to pay money back at a later date.

“I’m afraid it might make me lose other benefits or I might be asked to pay it back if it turns out I’m not eligible.” Mother, ID6005.

Other related concerns families have expressed include negative past experiences with officials or statutory services, leading to wariness about sharing personal information, and a lack of trust in authority.

“When I first applied for HS [in Summer 2022] NHSBSA asked me for the same information more than ten times, and I was asked to send the same documents again and again. I started to worry where all this data about me and my kids was going as they kept asking for it.” Mother, ID6004.

“I don’t want people to know my circumstances and personal details. I don’t really trust anyone.” Mother, ID7037.

##### Reframing HS as not a “benefit”

The framing of HS has been raised across stakeholder groups as a barrier to uptake. Some families experience shame or stigma in accepting schemes like HS which are considered to be “handouts” or “benefits”. Reframing the scheme as an entitlement or human right for childhood development has been suggested.

“People’s circumstances change, and they might need to take benefits, they should not feel ashamed, but they feel other people think ‘I am lazy because I’m on benefits’.” Mother, ID6004.

“Can we reword things so that people understand this is for them to help feed their children and they are entitled to it.” Local Authority, ID5008.

##### Families need a helping hand to apply

All stakeholders reported that many families need a helping hand to successfully register and claim HS payments. Many families need to work with someone to help them with the application process. Stakeholders noted that families with English as an additional language, low digital literacy or more challenging application processes (i.e. teenage mothers) need extra encouragement and support. In addition, professionals working with families noted that if an application is rejected, most families give up because no reason is given so they do not know if it can be rectified and reapply.

“The application is too long or too difficult for some people. They think it’s too much information they have to put in, usually doing it on their phone and it’s such a small screen, they think it’s formal and it puts them off; some of our families don’t have the literacy or the digital skills and they get put off very easily.” Local Authority, ID5032.

“They need to give an advice line so someone can help explain the reasons [if you are unsuccessful].” Mother, ID7028.

#### It is not always clear who can apply

Across stakeholder groups, limitations to the HS eligibility criteria were highlighted.

##### The eligibility criteria are too complex

Most stakeholders cannot understand the eligibility criteria for HS. These complex criteria result in it not being easily communicated or understood. Significant amounts of time and resources are spent trying to “demystify” who is, and who is not, eligible for HS. Many stakeholders expressed concern that the income threshold has remained stable for many years and now, during the cost-of-living crisis, families experiencing food insecurity are not eligible.

“I would have given up if the midwife I happened to meet at a local baby event had not intervened on my behalf. The refuge workers tried, the Sure Start centre people tried, but no one could help me until there was a personal contact at NHSBSA via this midwife. This is definitely not a robust or fair system.” Mother, ID6004.

“It’s just too complicated, it should be much simpler criteria. Frontline staff are so swamped they shouldn’t have to be struggling with this. We are constantly trying to demystify Healthy Start to frontline staff.” Local Authority, ID5028.

“The income limit is appalling, I do think it’s appalling, even the real living wage is more than that.” Local Authority, ID5033.

##### The system is unfair for very vulnerable groups

Applications for HS from those not in receipt of Universal Credit (UC) are made by paper applications because automated data checks are not possible. Stakeholders perceived this situation as being unequal, and potentially exacerbating health inequalities, particularly for teenage mums aged less than 18 years and asylum seekers. Some asylum seekers with No Recourse to Public Funds (NRPF) are eligible for the HS temporary ex gratia scheme which also requires detailed paper applications.

“There are so many young parents being denied HS and they are entitled to it, the paper application system for them is not working.” Local Health Professional, ID5013.

“There is not a clear understanding about the criteria for asylum seekers and those with no recourse to public funds (NRPF). I get so many queries from frontline staff. Everyone is confused and it’s so complicated—people just apply to flag the desperate need to government for this group.” Local Health Professional, ID5025.

##### Make HS an opt-out scheme

Across the system stakeholders mentioned that HS take-up rates would be optimal if the scheme were opt-out rather than opt-in. Although financial regulations currently hinder introduction of such an approach, the primary rationale stakeholders raised for an opt-out system was the fact that the families most likely to be missing out on HS are those with the greatest need.

“The biggest barrier to accessing Healthy Start is having to apply in the first place. It’s unnecessary and wastes a huge amount of everyone’s time, energy and resources that could be better spent elsewhere.” Local Authority, ID5028.

“The idea of Healthy Start is fantastic, but we would prefer our families to get automatic access instead of having to apply for it because we have a lot of digital exclusion, a lot of our families live chaotic lives, they have not great literacy. For the families we work with it’s just a step too far for them.” Local Health Professional, ID5039.

#### There is a disconnection of services at local and national levels

Families frequently reported that services to support them were not linked, making it difficult for them to access the schemes to which they are entitled. Professionals and retailers also mentioned a lack of joined‐up approaches for HS.

##### There is no‐one to talk to

Families noted that services are frequently being delivered online and that they would prefer to have someone on‐hand to assist with HS applications and queries. Families and professionals reported that the NHSBSA helpline has been described as helpful but delays in resolving cases can deter families. Having a named regional contact would help to optimise uptake.

“We need a BSA regional point of contact who can troubleshoot individual cases, someone who knows the system and works with us to get claims through.” Local Health Professional, ID5013.

“There is much less support now compared with back in 2008, there is a lot less contact with health professionals and especially after the birth they didn’t help enough, you need that support [for schemes like HS] but it is not there.” Mother, ID6003.

“Since the pandemic everything has become a bit faceless.” Local Authority, ID5008.

##### Many missed opportunities

Stakeholders noted that there are multiple opportunities to get families signed up to HS that are not currently coordinated or systematised.

“Why can’t the Job Centre use the Universal Credit journal to tell people about HS?” National NGO, ID5004.

“Why can’t new parents be told about HS when they register the birth of a child?” Local Authority, ID5005.

“Families come into contact with different services many times from pregnancy to when a child turns 2 years old, if these touchpoints were mapped and coordinated families would not miss out.” Local Authority, ID5017.

##### Government departments are not joined up about HS

Stakeholders perceived that a number of challenges in signing families up for the HS payment card could be overcome if cross‐departmental communication and data sharing arrangements were strengthened. Departments considered particularly important include Department of Health and Social Care (DHSC), Department for Work and Pensions (DWP), NHS Business Services Authority (BSA), Department for Levelling Up, Housing and Communities (DLUHC), His Majesty’s Revenue and Customs (HMRC) and Department for Education (DfE).

“All the government organisations are working in isolation and if they would work together more we could probably pick up all the people that are missing out.” Local Authority, ID5032.

“Why can’t DWP send us a list of those eligible for HS just like DfE sends us a list of the eligible 2 s, that would allow us to get everyone signed up so easily.” Local Authority, ID5034.

#### Capacity and resources are lacking

Across stakeholder groups there was acknowledgement that capacity and resources at the local level were limited. This situation hindered the level to which HS could be promoted and a helping hand could be offered to families on the ground.

##### Our workforce has been depleted

Stakeholders frequently commented on the depletion of local workforce as a major barrier to HS uptake. The vital role of the voluntary sector in helping families access and use HS was mentioned across stakeholder groups. There was concern that these organisations may themselves be struggling with the rising costs of overheads and this would further negatively impact families.

“The drop off in uptake can be tracked to the reduction in resourcing for early years services through austerity.” National NGO, ID5002.

“There have been extensive cuts and nationally there are not enough health visitors, we had a big call for action about 10 years ago, 1000s were trained and recruited, but we now have fewer health visitors than we had then due to cuts, changing structures, retirement, changed workload after Covid etc.” National Health Professional, ID5004.

“Voluntary sector organisations themselves are feeling very vulnerable due to rising energy costs and other increases. They tell us they might not be able to survive, and this really worries us because we have such a vibrant and dynamic voluntary sector in the city. This is a huge risk to families and could affect them significantly.” Local Authority, ID5009.

##### Make HS part of everyone’s job

In recognition that professionals are stretched in their current roles, several stakeholders suggested that HS should be incorporated into the role of everyone who has contact with pregnant women and young families. This approach would see professionals working in health, education, housing, childcare, social care and other services all supporting HS uptake.

“Everybody is struggling with capacity, so it needs to be everybody’s business and not one person’s responsibility.” National Health Professional, ID5003.

“We can’t expect the midwife to be all singing, all dancing, knowing about everything, because we haven’t got the capacity to do that, even though we would love to be able to do that, we just don’t have the capacity.” Local Health Professional, ID5013.

##### There are conflicting priorities

HS was noted as regularly not being the top priority when working with families living in complex contexts. Families eligible for the scheme may be facing homelessness, domestic violence, poor mental health or debt difficulties which need to take precedence over supporting an HS application.

“We want to do better but we have to look at what is the highest priority with any family that comes in the door. The Healthy Start application is a huge task – maybe we do short promotions, say 3 per year in order to really focus on getting families signed up.” Local Authority, ID5018.

#### Stronger leadership and accountability are needed

A number of stakeholders recognised that stronger leadership was needed to increase accountability and optimise uptake levels and benefits for families. This leadership is needed at local and national levels.

##### We have no clear lead locally

Stakeholders observed that in most communities it is not clear who is responsible for HS. Many people are working very hard to promote the scheme or train others to help families apply, but this work is not centrally coordinated, funded or evaluated, and often results in both duplication of effort and families missing out.

“Healthy Start should be everybody’s business, there are many professionals that come into contact with families, if it was on everyone’s KPIs then we would all be accountable.” Local Authority, ID5008.

“Currently there is no accountability for getting a family onto HS because it’s not clear whose job it is and I might think the midwife will do it but she might think I’m doing it, because it’s not anyone’s actual responsibility.” Local Health Professional, ID5039.

##### Little national leadership on implementation

Across stakeholder groups, there was recognition that local implementation of this national scheme was not clearly supported by national leadership or dedicated resources. This situation results in activities to support HS often taking the form of add‐on efforts rather than part of core responsibilities or coordinated action plans.

“Targeted promotion of the scheme in certain geographic areas where uptake is low – this is an easy ask to retailers. If we all act together in a specific postcode or local authority, everyone would be up for that.” National Retailer, ID5021.

“HS don’t provide promotional resources or materials for free. We don’t have a budget for printing, we don’t have colour printers either. A digital promotional pack is not good enough.” Local Authority ID5017.

“It was hard to engage with the team running HS and I don’t think they understood clearly the reach that we have as retailers, that was a bit of a barrier to the digitalisation which was a shame.” National Retailer, ID5050.

### Aim 2: To identify stakeholder recommendations to improve effectiveness and take-up of HS in England

This rapid qualitative analysis identified seven recommendations for consideration and further exploration. These recommendations reflect data from across all five stakeholder groups and aim to help improve uptake and effectiveness of HS in England. Detailed descriptions of these recommendations alongside stakeholder quotes illustrating their development are shown in Table 2 below.

**Table 2:** Seven policy recommendations based on stakeholder views

Policy recommendation	Illustrative quote
1. **Continue to offer Healthy Start to families and assess how much the current value enables families to purchase**	
Healthy Start is an extremely valuable government food policy, unanimously supported by all stakeholders. The overall value is seen to offer less than in the past due to increasing food prices	“My view of Healthy Start is overwhelming positive, make no mistake, this scheme makes a real, tangible and significant difference to household food budgets, as well as to the dietary intake of young children.” National NGO, ID5040
	“It’s a fantastic benefit and if only it could be in line with inflation. Nowadays it doesn’t even come close to covering the cost of formula or enough fruit and veg for a week.” Local Authority, ID5036
	“Healthy Start is a life saver, but since the cost of living it just doesn’t cover anything anymore” Mother, ID7016
2. **Establish a solution-driven, cross departmental Healthy Start specific working group**	
Develop a solution-driven cross departmental working group for HS (including DHSC, NHSBSA, DWP, DLUHC, HMRC, His Majesty’s Treasury, Cabinet Office) to explore and coordinate activities such as data sharing, touchpoints across statutory services to flag the scheme to eligible families, options for opt-out implementation and extending provision until a child starts school	“All the work that is done like training local professionals and HS Champions, the impact on uptake is marginal. It is smaller than we would like. This is the key point to Government, here we are putting all the blood, sweat and tears into promoting Healthy Start and helping families to apply, but still there is only a marginal increase, because local action is no substitute for national policy and coordination.” National NGO, ID5040
	“Healthy Start should continue until the child starts school, it’s a no brainer in terms of childhood development and where is the evidence that nutrient requirements change when a child turns 4?" Local NGO, ID5044
	“It should not be a fighting battle to get Healthy Start. It should be automatic via auto-enrolment for eligible families.” Local Authority, ID5028
3. **Reframe the language surrounding Healthy Start to co-create appropriate and inclusive wording**	
It is important to work with a wide range of families to develop wording to promote and describe Healthy Start that is inclusive, empowering and enables everyone to feel comfortable talking about the right for children to access a healthy diet and develop well	“It’s all part of the messaging campaign, it’s about how it is framed, you know, 'You have a right to this, to feed your children, it won’t affect your other payments, you meet the eligibility criteria.’ Some clarity around this message is needed.” National NGO, ID5040
	“White British families are accessing this support much more than ethnic community populations. We need to work better with community leaders and groups to talk about HS more, through trusted relationships, to move families forward with it. Find out what the barriers are for those communities.” Local Authority, ID5008
	“People feel they don’t want to take more from the government who have already given so much and therefore they don’t apply out of shame or pride.” Local Authority, ID5028
	“Parent champions are essential for taking the Healthy Start message back to the Bangladeshi and Chinese communities.” Local NGO, ID5044
4. **Provide national leadership on a Healthy Start promotional campaign, ensuring coordinated activity and adequate resourcing for providing families with a helping hand to complete the application**	
National leadership on a promotional campaign is needed to coordinate action by public, private and voluntary sector stakeholders. Adequate resources are needed for local services to provide a helping hand to family through the application process and resource promotional activities	“National leadership and investment in promotional campaigns are needed, to bring together all the local stakeholders including the public, private and voluntary sectors.” Local health professional, ID5013
	“We know hand-holding works well for a lot of our complex families but we don’t have the resources to do that.” Local Authority ID5034
	“Job centre staff don’t have capacity to help people fill out the application form, they would ideally signpost families to local services that can help with that, but unfortunately due to lack of capacity in local statutory and voluntary sector there is limited support to do this.” Local Authority ID5053
	“Our workers come from the communities that they work in. They are having conversations with families all the time in many different settings including local parks.” Local Authority ID5036
5. **Hold regular and coordinated three-way communications between national, regional and local services**	
Regular communication between national, regional and local services would assist the flow of information, concerns and successes top down and bottom up. OHID regional teams could coordinate communication between stakeholders including local DWP teams, Local Authority teams such as Early Years, Children and Young People, 0–19 services, local voluntary sector organisations, and NHSBSA and national DHSC policymakers. Additional resource for these activities may be necessary	“Let’s not reinvent the wheel, let’s use existing structures like OHID regional teams to communicate about Healthy Start in a bottom up as well as top-down manner.” Local Authority, ID5012
	“We need regular meetings with NHSBSA to start up again with open dialogue between us and them. We don’t always understand what the application problems are so if they could share more about that then we would know what to do to fix them.” Local Health professional, ID5013
	“The people at NHSBSA are very helpful and friendly but are clearly hugely under-resourced.” Local Authority, ID5038
6. **Coordinate local action, potentially through Health and Wellbeing Boards**	
Health and Wellbeing boards offer one mechanism for ensuring coordinated local action, monitoring and accountability, particularly if made statutory	“Local Health and Well-being Boards or their equivalent could lead a Healthy Start Steering Group with all relevant services as members including health, housing, education and others, to develop action plans, coordinate activities and monitor progress.” Local Health professional, ID5013
	“Our research locally showed that no one had Healthy Start in their job role, so that was one of our main recommendations and now it is officially in the job role of a member of the local public health team. She coordinates the HS working group and is the focus point for coordinating action across the city, but we need more leadership and accountability to make a real difference.” Local NGO, ID5044
	“Give Healthy Start dedicated Council time in the debating chamber once a year perhaps to report on progress and debate why haven’t we achieved our target?” National NGO, ID5040
7. **Develop local “one-stop-shops” that are adequately resourced to support all families holistically**	
Families want a single place to go in their local communities where they can access a range of services, including a helping hand with Healthy Start applications. Community centres which are universal and recruit local people have been life-changing for some parents and families. Outreach facilities are necessary in rural areas and to reach the most vulnerable families not opening mainstream services	“We need more community-based spaces for families to access, and they must be open to all families, because if targeted then the intended families don’t go. These spaces are also important for modelling behaviour, e.g. how to read with your child, how to cook healthy foods.” Local Authority ID5008
	“My daughter is going to uni now which I would never have believed ten years ago, but because of Sure Start I was able to deal with a lot of my mental health issues [….] and the help I got made me into the person I am now, working here and helping others like me.” Mother, ID6006
	“We have been able to work together with our local DWP Partnerships Manager to make sure all Job Centre staff know about HS and are able to talk to new suitable UC claimants about it in their Commitments Meeting. Thanks to this partnership working, all local DWP Work Coaches should understand what HS is and be able to advise families if they might be eligible or not.” Local Authority ID5053
	There are some families and some communities we just can’t reach, it’s about more than just awareness and training professionals, we need outreach workers, we need resources to do that, we need people from the community to reach out because of the trust issue. Local Authority ID5038

The two recommendations for immediate consideration are as follows: (i) continue to offer HS to families and assess how much the current value enables families to purchase and (ii) establish a solution-driven, cross departmental HS-specific working group.

Three recommendations are more intermediate as they require participatory work to ensure effectiveness; these include the following: (iii) reframe the language surrounding HS to co‐create appropriate and inclusive wording, (iv) provide national leadership on a HS promotional campaign, ensuring coordinated activity and adequate resourcing for providing families with a helping hand to complete the application and (v) hold regular and coordinated three‐way communications between national, regional and local services.

The two remaining recommendations require longer-term action and possibly legislative changes to enable enaction, namely: (vi) co‐ordinate local action, potentially through Health and Wellbeing Boards and (vii) develop local “one‐stop shops” that are adequately resourced to support all families, including resources for outreach work.

## Discussion

This study is timely in its overlap with the current cost-of-living crisis and assessment closely following digitalisation of the HS scheme. The findings demonstrate that HS makes a valuable difference to families’ abilities to purchase healthy foods for their young children. The payment card is considered preferable to the paper vouchers for the vast majority of families because it is easier to use in stores and the credit can be accumulated for bulk purchases. The application process, however, can be challenging for many families.

Several key factors are likely to be driving lower than expected take-up rates. Stakeholders spoke about the lack of coordinated action regarding HS at local and national levels leading to a disconnect between departmental activity and service provision. This situation makes it difficult for families and local workers to know who to turn to for timely support regarding uncertainties in eligibility or the application process, which is not currently universal for all families.

New insights revealed that awareness of HS is not sufficient to increase take-up. Where possible, local areas have employed huge local resource, often funded by the voluntary sector, to actively promote HS through flyers, posters, stickers and other activities such as training frontline staff. Yet, personal values, cultural beliefs and family dynamics can prevent families signing up. Reframing the language surrounding HS by working with a diverse group of eligible households is likely to help inform future promotional work. To achieve greatest impact on take-up rates, however, promotional activities need to be coordinated and coupled with adequate resourcing so families can be offered a helping hand through the application process. A policy-specific, cross-departmental working group that acts horizontally (at both national and local levels) and vertically would facilitate accountability on uptake rates, efficiency of implementation and identification of opportunities to simplify registration with the aim of optimising uptake.

### Comparison with previous research

In addition to adding unique and valuable insights to the evidence base, findings from this study support existing evidence about HS. Overwhelmingly, stakeholders spoke about the importance and tangible value HS brings to families. Similar to investigations of the previous paper-based HS voucher, parents described how the payment card resulted in meaningful increases in the quality and range of healthy foods they could access for their young children, particularly fresh fruit and vegetables [5, 6, 15, 32, 33]. They also noted that HS allows their children to establish healthy dietary preferences at an early age, helping them to develop good food practices for the future [6]. Previous assessments of HS in England reported that families had trouble using vouchers in their local convenience stores or at more affordable market stalls because retailers were required to register and follow arduous reclaim processes [6]. Digitalisation has largely overcome these barriers, with the HS payment card being accepted in all outlets recognised as food retailers, including market stalls and community supermarkets which are set-up to accept Mastercard™. Most families appreciate the increased freedom and reduced stigma associated with the payment card.

Stakeholders raised concerns about who can obtain the HS card and its low monetary value which echoes previous work [6, 34, 35]. The primary issues raised across stakeholder groups taking part in this study included the (i) value not keeping up with rising food costs, (ii) gap in nutritional support between a child’s fourth birthday and starting school, (iii) income cut point being lower than the poverty line, (iv) complexity of the eligibility criteria, (v) differences in the application process for certain groups and (vi) opt-in nature of the scheme. UK Members of Parliament have previously raised a number of these concerns during Parliamentary Question and Private Members’ Bill sessions in 2022 and 2023 [36, 37]. DHSC Ministers have responded that the UK Government and NHSBSA (who operates HS) are committed to increasing uptake of the scheme. They have also highlighted the requirement that each family meeting HS eligibility criteria must accept the conditions of the payment card and that it is this legal requirement for financial products like the HS payment card which prohibits it being issued automatically to eligible families [36].

The creation of a solution-driven cross-departmental working group specifically for the HS scheme was called for by a number of stakeholders. Despite several existing cross-departmental working groups covering broad topic areas such as poverty, development of a HS-specific working group would act to efficiently connect actors, activities and data spread across relevant national government departments including DHSC, DWP, HMRC and His Majesty’s Treasury and Cabinet Office. Input from across these departments could help to systematically address each of the concerns outlined above and coordinate promotional activity by retailers. Policy-specific cross-departmental working groups have been identified as an important mechanism for achieving food policy coherence and effective policy implementation across government [38]. Senior leadership is recognised as being a necessary component of cross-department groups so that collaborative working is encouraged, recognised and rewarded, as well as evaluated as part of the evidence collected to assess policy effectiveness [39]. Development of a HS-specific cross-departmental working group could act to address current policy priorities across UK government departments on reducing inequalities and food poverty, particularly during the current cost-of-living crisis [40]. Immediate action to review the amount of food the HS scheme’s current value enables families to purchase and mechanisms to simplify or expand the eligibility criteria would be welcomed by families.

### Policy and research implications

An important and novel finding from this study is that raising awareness about HS alone is unlikely to result automatically or universally in higher uptake rates, in part because eligible families have reported feeling that HS is not for them for reasons related to identity, culture and values. While knowledge of the HS scheme is not widespread across society [6, 14] and there are calls for a national promotional campaign [34, 41], reframing the language used to describe and promote HS could increase its perceived relevance to all eligible families. Several stakeholders noted the need to reframe the scheme as an entitlement or fundamental human right for healthy childhood development rather than a benefit. The UK Government has committed to ratifying the International Covenant on Economic, Social and Cultural Rights and is legally required to secure the human “right to food” for everyone in the UK [42]. The “right to food” is defined as being able to access sufficient food in a quantity and quality that satisfies dietary needs for physical and mental growth and for foods to be free from adverse substances and culturally acceptable [43]. HS could be positioned as one of the UK government’s mechanisms for supporting very low-income families with young children to meet this legal requirement. Working closely with a diverse range of families across England using novel qualitative and participatory research methodologies would provide valuable insight into HS reframing [44].

A crucial finding from this study is that many families need a helping hand to complete the HS application process. Without this support, promotional work targeting uptake cannot convert to significant increases in uptake figures. Many families eligible for HS are facing multiple challenges daily, such as financial and housing insecurity, poor-quality housing and managing illness or disability [8]. These contexts mean that successfully applying for HS remains an onerous and challenging task. Not being a native English speaker, having low literacy skills and poor digital access or competence present additional hurdles to successfully completing the online form [6]. Families who receive an unsuccessful notice upon their first application are unlikely to try again. Although families and stakeholders reported that NHSBSA’s helpline and resources to support families are helpful, many felt that not providing a reason to families for a rejected application can leave them confused, disillusioned and subsequently disengaged. Rejected applications occur because of typos, differences in spelling of addresses/family details when cross-checked with DWP records. Providing on-the-ground support to families while they complete a HS application makes the difference between a family accessing this funding to feed their children well or not.

This study showed that families with English language or digital access difficulties, who fear or mistrust authority, or who have poor mental/physical health, not only require one-to-one support but that it comes from a trusted individual and in words they can understand and relate to. In Blackpool, dedicated funding from the Big Lottery Fund allowed a team of “community connectors” to be recruited, trained and employed to work in the community to build trust and rapport with local families and support them to access services, including HS. Subsequently, uptake rates accelerated in this area to 72% [45]. This example and evidence from service improvement work targeting HS uptake elsewhere [46] illustrate the critical need for dedicated and coordinated staffing resource in communities to increase uptake of this highly valued scheme nationally. Future research to assess the costs of community outreach resources, particularly in areas with the lowest uptake rates, could provide valuable information for future HS budgetary estimates.

Several stakeholders reported a desire for more structured and regular communication between national, regional and local government agencies to learn about HS scheme updates, voice concerns and queries from their communities and share examples of best practice. Regional teams of the Office for Health Improvement and Disparities (OHID), with their priority to ensure a joined-up approach to delivering core services promoting healthy diet and child health [47], are well-placed to facilitate vertical integration on HS between central and local teams. Having regular representation from DHSC and NHSBSA at these regional meetings would facilitate sharing of best practice from local regions [48].

The need for co‐ordinated local action with a clear accountability framework was recommended by stakeholders to facilitate and sustain increases in HS uptake rates [49]. While Health and Wellbeing Boards have limited formal powers, they could be required to assess local HS uptake figures regularly and implement improvements should rates drop below a certain level as part of the UK government’s commitment to “the best start for life” and “right to food” [42, 50].

There are numerous examples of best practice on the ground which illustrate the unique position of health professionals to encourage HS uptake. For example, in Manchester midwifery services have embedded HS into training and clinical practice via the electronic patient pathway. Yet, the depletion of midwives and health visitors over the past decade, time pressures and competing priorities means responsibility for HS implementation cannot solely rest with health professionals. Rather, stakeholders expressed the need for HS to be “everyone’s business” and for better connection of local services ideally in the form of local “one‐stop shops” where services for child health, housing, job seeking, parenting etc. can all be accessed. There is hope that positive outcomes will result from the new Family Hub Programme being rolled out in 75 local authorities across England which could then lead to wider and sustained implementation [51].

### Strengths and limitations

This study brings together the perspectives and experiences of multiple stakeholder groups from across the HS system. This approach provides policymakers with a holistic view and novel insights which could be leveraged to optimise take-up of HS and bring benefit to more families. The sampling approach and two-phase methodology applied in this study allowed a wide range of participants to take part across England. Combining the Activmob approach with semi-structured in-depth interviews by scientific researchers provided concurrent collection of a rich qualitative dataset from which cross-cutting themes were elicited [27]. The recruitment approach may have introduced participant bias attracting stakeholders with strong opinions about HS and rural areas were not covered; however, the study sample included perspectives from different English regions, local and national stakeholders and various sectors within the HS delivery system.

A limitation with rapid qualitative evaluations can be having sufficient data on which to base decisions. The themes and recommendations developed from the data collected in this study, however, incorporate views from 112 participants and were consistent across all stakeholder groups and all research sites. Additionally, team-based reflexivity methodology for rapid qualitative health research was applied during analysis [52] and regular discussions about the findings with core teams from each case study site and the study’s public contributor panel provided validation and refinement of findings from the rapid analysis. A further limitation of this study is its focus on the payment card component of HS. Future research could explore perceptions and experiences of the free vitamin component of the scheme, including potential for better alignment with the payment card.

## Conclusions

This rapid qualitative evaluation has shown that HS delivers significant and much-valued support to some of the most economically vulnerable families in society. HS helps pregnant women and families to access nutritious food for themselves, their babies and young children that they otherwise would not be able to afford. Yet, despite significant promotional activity in local areas, many families continue to miss out on this nutritional safety net. An important and novel finding from this study is that raising awareness about HS alone is unlikely to result automatically or universally in higher uptake rates because many eligible families need a helping hand with the application process and some families feel HS is not for them for reasons related to identity, culture and values. Participants also felt that services were often disconnected on the ground. Recommendations identified by stakeholders to optimise HS uptake include: the provision of local resources for families to access a helping hand to successfully complete the application; reframing the scheme as a child’s right to food to maximise inclusivity and uptake; and improved leadership, coordination and accountability for the scheme at both national and local levels.

### Supplementary Information

**Additional file 1.** Interview guides for each stakeholder group.

## Data Availability

Data described in this manuscript that have been collected by the research team during this study, and that can be anonymised, can be made available upon reasonable request to the corresponding author pending approval.

## Abbreviations

DfE: Department for Education
DHSC: Department of Health and Social Care
DLUHC: Department of Levelling Up, Housing, and Communities
DWP: Department of Work and Pensions
HMRC: His Majesty’s Revenue and Customs
HS: Healthy Start
NGO: Non-governmental organisation
NHSBSA: NHS Business Services Authority
NRPF: No Recourse to Public Funds
OHID: Office of Health Improvement and Disparities
RAP: Rapid Assessment Plan
UC: Universal Credit
