# Supplementary material for: The Healthy Start scheme in England “is a lifeline for families but many are missing out”: a rapid qualitative analysis
Source: BMC Med. 2024 May 8;22:177. doi: 10.1186/s12916-024-03380-5 (PMC11077836; doi:10.1186/s12916-024-03380-5)
Supplement: Supplementary file 1 — Additional file 1. Interview guides for each stakeholder group. [file 12916_2024_3380_MOESM1_ESM.doc]

**HEALTHY START EVALUATION**

**DISCUSSION GUIDE FOR FAMILIES**

**Introduction checklist**

1. Participant Information Sheet (to cover rationale, objectives, who is being involved and why, outcomes)
2. Clarify reasoning and use of the project and this conversation- anonymous and confidential
3. How will the discussion be structured- capturing demographics
4. Consent form

*(Note: the Healthy Start card was introduced in November 2021 and vouchers were phased out completely 31^st^ March 2022)*

| **TOPIC and PROMPTS** | **NOTES** |
| --- | --- |
| 1. **About you**   **HEALTHY START SCREENING QUESTION** e.g. do you currently receive HS support? |  |
| **2. Knowledge of Healthy Start**  **What do you know/have you heard about the Healthy Start Scheme?**  **How do you feel about Healthy Start?**   - How has this opinion changed over time? How and why? - What extra information do you need? - Have you tried to find out anything? What/how/where?   How well-informed do you feel about the Healthy Start scheme? Score 1 – 5 (5 being fully informed)  **How/When did you first hear about Healthy Start?**  **Who/what did they say?**  **Map start of journey** |  |
| **3. Accessing Healthy Start (continue mapping)**  **What happened when you got/offered Healthy Start?**   - What happened first? - How did you decide to access the scheme or not? - How did this decision change along your journey? For what reasons? - How did you find the process of accessing the scheme? - What barriers did you face? - How did you manage these? - What made it easy to access the scheme? - How did you feel at each step in the process? - How have you found it now it is digital? - What were the differences in accessing the vouchers/card/vitamins? |  |
| 1. **Using Healthy Start (continue mapping)**   How did you find the process of using the vouchers/card?  What do you know about where to use the HS card/vouchers?  What difficulties have you had using the vouchers/card?  How did this make you feel?  What does a typical time that you have used HS vouchers or the card look like?  How did it work?  What were the highs/lows  What kinds of things do you buy with it?  How do you decide?  What factors influence what you buy?  What things do you need but can’t buy with the card e.g. dairy alternative milks for babies/toddlers with allergies  What differences have you found using the card compared to the vouchers?  **Explore factors in the store e.g. price, promotions, availability, as well as other factors like pressure from kids/partner/friends** |  |
| 1. **Financial value and vitamins**   What do you think about the amount of money offered in HS?  The value increased from £3.10 to £4.25 per week in 2021, how did this impact on what you buy?  How often have you accessed the HS vitamins?  How is that process different to accessing the vouchers/card?  What do you think about who can and those who cannot apply for the HS scheme?  What do you think about the age of children that are eligibility for HS? |  |
| 1. **Future**   What three things do you think the government could do to encourage you/others to access and use Healthy Start prepaid cards?  Probe around   - Information- who/what/where/when - support with decision-making - changes to access (including age range) - digitalisation |  |
| 1. **Add-on services**   What add-on services have you been offered alongside the HS scheme?  What add-on services or activities would you like to be offered as part of the Healthy Start scheme and who do you think should offer them? For example:   - *cooking skills/healthy eating classes* - *accessing cooking equipment* - *cooking fuel schemes* - *educational resources* - *special promotions for HS users by retailers etc)* |  |

**THANK YOU FOR YOUR TIME TODAY**

**HEALTHY START EVALUATION**

**DISCUSSION GUIDE FOR PROFESSIONALS**

**charities, NGOs, community organisations, health and social care professionals**

**Introduction checklist**

1. Participant Information Sheet (to cover rationale, objectives, who is being involved and why, outcomes)
2. Clarify understanding of rationale for evaluation of HS
3. Clarify process for anonymity and confidentiality
4. How will the discussion be structured- capturing demographics on separate form
5. Consent form and permission to record interview

*(Note: the card was introduced in November 2021 and vouchers were phased out completely 31^st^ March 2022)*

| **TOPIC and PROMPTS** | **NOTES** |
| --- | --- |
| 1. **About you**   Name  [Brief description of duties] |  |
| 1. **Eligibility and scope of HS**   What is your general feeling about the HS scheme?  What is your understanding of the eligibility criteria of HS?  How easy is it to identity families who are eligible?  What do you think about the income criteria?  What do you think of the child age criteria?  What do you think about the value of financial support offered in HS?  What do you think about the items families can buy with the HS financial support? |  |
| 1. **Uptake and promotion**   How do you receive information/learn about the details of the HS scheme?  What communications and/or promotional materials do you use to raise awareness of the scheme?  What tools or support would be useful to improve uptake and awareness in the scope of your role?  Who else do you know that promotes the HS scheme and how linked up with them is your organisation around this?  In your opinion, who else should be promoting the scheme and how could this be done?  How do you/your organisation monitor uptake to the scheme? What level of area/locality is of most interest to you in terms of uptake figures? |  |
| 1. **HS add-ons:**   What add-on services do you/your organisation offer alongside the HS scheme?  What add-on services or activities do you think could/should be offered as part of the Healthy Start scheme, and who should deliver them?  - *cooking skills*  *- accessing cooking equipment*  *- cooking fuel schemes*  *- educational resources*  *- special promotions for HS users by retailers etc* |  |
| 1. **Use and access**   How easy is it to refer families to the scheme?  What types of families access the scheme in your area?  What types of families do you feel are least likely to access the HS scheme?  What training is available to you/your colleagues to promote the HS scheme to eligible families?  What are your experiences of supporting families to access/apply for the scheme? What differences are there for vouchers/digital card/vitamins?   - *Language* - *Eligibility criteria/ NRPF* - *Digital access* - *Processing time* - *Technical difficulties with digital process*   What do you know about how families use the vouchers/card/vitamins?  Where do people mostly spend the funds? e.g. retailer, market, food banks/pantry  What do you know about how retailers are reimbursed from the government?  How important is it for you to know these details? |  |
| 1. **HS impact**   How well do you think the HS scheme supports eligible families to healthily?   - *quantity vegetable/fruit purchases?* - *Better variety / quality vegetables/fruit?* - *Reduction in unhealthy foods?* - *How well do you think the HS scheme helps families to sustain any change in habits?*   In what other ways do you think the HS scheme helps families?   - *reduce food bills* - *reduce stress/worry* - *reduce food insecurity*   How helpful do you think the HS scheme is for families right now during the cost of living crisis and pressures of inflation? |  |
| **Other thoughts**  What research has been done in your local area/by your organisation about HS promotion, uptake, use or impact?  What else would you like to say that has not already been covered in this discussion? |  |

**THANK YOU FOR YOUR TIME TODAY**

**HEALTHY START EVALUATION**

**DISCUSSION GUIDE FOR RETAILERS**

**Introduction checklist**

1. Participant Information Sheet (to cover rationale, objectives, who is being involved and why, outcomes)
2. Clarify understanding of rationale for evaluation of HS
3. Clarify process for anonymity and confidentiality
4. How will the discussion be structured- capturing demographics on separate form
5. Consent form (including RECORDING)

*(Note: the card was introduced in November 2021 and vouchers were phased out completely 31^st^ March 2022)*

| **TOPIC and PROMPTS** | **NOTES** |
| --- | --- |
| **About you:**  Name  [Brief description of duties] |  |
| 1. **Eligibility and scope of HS**   What is your understanding of the purpose of the HS scheme?  What do you think about the income and child age criteria?  What do you think about the financial value of HS and the products families can buy with this support? |  |
| 1. **HS promotion and uptake**   How important is HS to your company?  How long has your company been registered with the HS scheme? How did digitalisation change this?  What ways does your company promote the HS scheme to customers who are eligible? What has been done in the past?  What impact do you think these promotional activities have had?  How willing do you think your company would be to offer an uplift in financial value? What would this look like?  How has digitalisation of the scheme changed the way your company supports the HS scheme? |  |
| 1. **Use of HS card/vouchers**   How easy is it for customers to use the HS card in your stores? How does this differ from the previous vouchers?  What products are customers buying with the HS card? How does this differ to the vouchers?  What impact do you think the HS scheme has on customers’ overall shopping basket in your stores?  How easy is it for your company to administer the HS scheme? How has this changed with digitalisation?  How easy is it for your company to claim for reimbursement from the government? How has this changed with digitalisation? |  |
| 1. **HS impact**   How effective do you think HS scheme is at achieving its aims (healthy eating)?  What impact do you see the HS scheme has on customers in your stores?  How helpful do you think the HS scheme is for families right now during the cost of living crisis and pressures of inflation?  What research has been done by your company about HS promotion, uptake, use or impact?  What else do you think government or professionals could do to enhance the impact of the HS scheme?  What ways is your company supporting/advocating these activities?   - *rediverting funds to support charities who can promote the scheme* - *advocacy/conversations with Government?* |  |
| **Other thoughts**  What research has been done in your local area/by your organisation about HS promotion, uptake, use or impact?  What else you would like to comment on in relation to the HS scheme, from a retailer’s point of view? |  |

**THANK YOU FOR YOUR TIME TODAY**
